# A Probabilistic Method for Estimation of Bowel Wall Thickness in MR Colonography

**DOI:** 10.1371/journal.pone.0168317

**Published:** 2017-01-10

**Authors:** Thomas Hampshire, Alex Menys, Asif Jaffer, Gauraang Bhatnagar, Shonit Punwani, David Atkinson, Steve Halligan, David J. Hawkes, Stuart A. Taylor

**Affiliations:** 1 Centre for Medical Image Computing (CMIC), University College London (UCL), Gower Street, London WC1E 6BT, United Kingdom; 2 Centre for Medical Imaging, University College London, 250 Euston Road, London NW1 2PG, United Kingdom; North Shore Long Island Jewish Health System, UNITED STATES

## Abstract

MRI has recently been applied as a tool to quantitatively evaluate the response to therapy in patients with Crohn’s disease, and is the preferred choice for repeated imaging. Bowel wall thickness on MRI is an important biomarker of underlying inflammatory activity, being abnormally increased in the acute phase and reducing in response to successful therapy; however, a poor level of interobserver agreement of measured thickness is reported and therefore a system for accurate, robust and reproducible measurements is desirable. We propose a novel method for estimating bowel wall-thickness to improve the poor interobserver agreement of the manual procedure. We show that the variability of wall thickness measurement between the algorithm and observer measurements (0.25*mm* ± 0.81*mm*) has differences which are similar to observer variability (0.16*mm* ± 0.64*mm*).

## 1 Introduction

### 1.1 Motivation

Crohn’s disease (CD) is an important healthcare problem which affects 700,000 people in the USA and 500,000 in Europe [1, 2]. CD manifests as a relapsing inflammatory disease that mainly affects the gastro-intestinal tract, producing disabling symptoms including abdominal pain, diarrhoea and vomiting, weight loss, loss of appetite, arthralgias (joint paint), fever, and fatigue [3]. Management of CD is multi-disciplinary and revolves around immunosuppressive therapy and judicious use of surgical resection. The disease has a lifelong relapsing course with periods of acute inflammatory activity interspaced with periods of relative quiescence [1, 3]. The key to successful management is correct identification of the active phase such that appropriate treatment with immunosuppressive medication can be instigated and remission sustained. Such medications however attract a significant side effect profile and not all patients respond. Another important facet of disease management is therefore monitoring of therapeutic effect such that treatment regimens can be changed as appropriate. Identification of active disease and morning of therapeutic effect in CD is problematic [1]. Arguably the gold standard is endoscopy which can visualise the mucosa of the colon and terminal ileum. However the test is highly invasive, expense, and cannot assess extra luminal complications or the proximal small bowel. Blood and stool tests such as CRP and faecal calprotectin have a role but lack both sensitivity and specificity. Cross sectional imaging, particularly MRI is increasing using to diagnose relapse in CD and monitor therapeutic interventions [4]. In the case of MRI, the test is non-invasive and does not impart ionising radiation. Furthermore it is able to assess both luminal and extra luminal disease including abscess and fistulation [5]. Various parameters on MRI have been validated as biomarkers of disease activity against both endoscopy and histopathology. Notably bowel wall thickness, T2 weighted mural signal and contrast enhancement on post gadolinium T1 weighted images are highly correlated with inflammatory activity [6, 7]. MRI activity scores have been derived which include these parameters. For example the Magnetic Resonance Index of Activity (MaRIA) score is highly correlated with an endoscopic activity score (Crohn’s Disease Endoscopic Index of Activity) and includes evaluation of all these MRI features as well as ulceration by the reporting radiologist [8]. Similarly, Steward et all developed an MRI activity score using surgical resection specimens and subsequently validated it against endoscopic biopsy [9]. Again the main facets of the scoring system are bowel wall thickness, T2 mural signal and contrast enhancement. Importantly, this score has recently been show to accurately reflect therapeutic response [10]. Such scoring systems require good inter and intra observer variation. Furthermore they are time consuming to apply in clinical practice. Measurement of bowel wall thickness is a vital part of activity scoring but is subject to inter-observer variation and is particularly time consuming, requiring manual placement of electronic callipers by the reporting radiologist. Automation of bowel wall thickness measurement to improve accuracy and efficiency is therefore highly desirable.

### 1.2 Related Work - Technical

The need for a computer-assisted model for automated detection of mural inflammation in patients with Crohn’s disease was identified in [11]. The ability to grade disease severity is recognised as limited due to the weak to moderate interobserver variability of subjective MRI features [12]. A pipeline for automated assessment of disease severity was introduced by [13], including procedures for image analysis, classification and visualisation to predict colonoscopy disease score. Included is a proposal of a bowel wall segmentation method to allow analysis of bowel wall thickness as a function of time; a method for registration of Dynamic Contrast Enhanced MRI (DCE-MRI) images, to allow for generation of accurate Time Injection Curves (TIC); a method for identification of diseased regions by classification of texture features by machine learning techniques; and finally a method for effective visualisation of disease by volume rendering.

In [14] low level features based on image intensity, texture and shape asymmetry are combined with a supervised learning approach to classify patches of pixels in abdominal MRI images as ‘diseased’ or ‘normal’. Texture maps are created based on Gabor filter banks, and shape asymmetry features based on entropy of orientation distributions with each patch providing multidimensional features. A range of classifiers are used: random forests, support vector machines, and a naive Bayesian classifier giving a high level of classification accuracy. [15] extend this method to perform efficient classification over a large volume, by first segmenting the volume into super voxels by a k-means clustering of voxels using image boundary information [16]. This allows random forest classification of supervoxels followed by graph cut segmentation to identify contiguous regions of disease. Most recently in [17], feature vectors are extracted from overlapping 8 × 8 voxel image patches. A query sample selection helps select the most informative unlabelled patches by visual saliency, allowing a radiologist to classify the patches with the most contextual information. Again, graph-cuts are used to segment the volume based on the image patches to classify areas of disease.

[18] propose a method to perform non-rigid motion correction in free-breathing abdominal DCE-MRI data. A large number of volumes are dynamically acquired and retrospective gating used to select those volumes which are in a similar point in the respiratory cycles, based on the sum-of-squared-differences error between them. A Discrete Cosine Transformation (DCT) [19] is then used to co-register all selected volumes to a reference volume. The effectiveness of the algorithm is evaluated by assessment of the TICs in three regions of interest, showing less fluctuation in the gated and registered images, in comparison to the entire dynamic volume stack.

A method for measurement of wall thickness of the colon using ultrasound has been published [20]. Multiple methods are evaluated for segmentation of a single image slice: Otsu’s thresholding [21], followed by region growing; level set segmentation; adaptive thresholding; Canny edge detection. The distance across these segmented region is measured and compared against a human reader. Results for a very small sample size (n = 5) are shown.

### 1.3 Proposed method

The following contributions are described in further detail in section 2:

- A novel method to automatically track sections of colon, and subsequently make measurements of thickness of circumferential loops of the bowel wall. A state distribution is used to model the bowel, with each state corresponding to a position, orientation and shape of the lumen. A Bayesian recursion equation is applied to estimate the posterior density of the state space, by repeating prediction and measurement steps based on the MRI volume, using a framework called particle filtering [22]. The resulting density is then used to calculate the centreline position. Although not applied to the bowel before, this framework has been used in the tracking of cerebral arterial segments [23].
- A novel method is proposed to estimate the positions of the inner and outer bowel wall by analysis of cross sections corresponding to the plane perpendicular to the centreline. The problem is modelled with a MRF, using gradient based metrics extracted from the MRI volume to assign costs to each possible bowel wall position. Performing inference on this model allows estimation of the optimal position of the inner and outer bowel wall, and therefore the thickness. This method is shown to give similar thickness measurements, and a similar level of inter-observer variability to the human reader.

## 2 Methods

### 2.1 Overview

**Fig 1:**
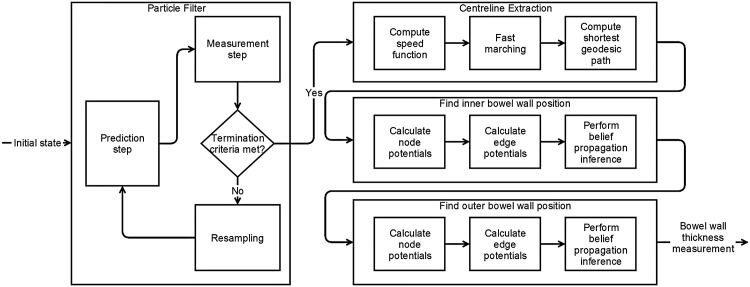
A flowchart providing an overview of the method to perform automated bowel wall thickness measurements.

As a first step, the bowel is tracked using the particle filter method described in section 2.2. The resulting particle information is used to extract a smooth centreline using the fast marching technique described in section 2.5.2. Finally, the positions of the inner and subsequently the outer bowel wall are computed using image gradient information and a MRF model using the method described in section 2.6. This overview is shown in Fig 1.

### 2.2 Particle Filter

The theory and algorithms in [22] can be used to estimate the state of a dynamic system given a sequence of noisy measurements. They are often used to measure the evolution of state over time, although any dynamic factor can be substituted. In the following section we propose to track the colon by evolving the state of a system containing the central position of the bowel as well as its orientation and cross sectional shape. Initially the shape will be modelled with an ellipse with varying radius and orientation. The assumption of smooth variation along the axis of the structure gives a good argument for the use of a tracking-based solution.

### 2.3 State Vector

The initial state of the system is modelled as a 15-D vector:

$$\begin{array}{ccc} x_{t} & = & {\left[c_{x_{t}}\;c_{y_{t}}\;c_{z_{t}}\;r_{11_{t}}\;r_{12_{t}}\;r_{13_{t}}\;r_{21_{t}}\;r_{22_{t}}\;r_{23_{t}}\;r_{31_{t}}\;r_{32_{t}}\;r_{33_{t}}\;a_{t}\;b_{t}\;\phi_{t}\right]}^{T} \end{array}$$ (1)

$$\begin{array}{ccc} & = & {\left[c_{t}^{T}\;r_{t}^{T}\;e_{t}^{T}\right]}^{T}, \end{array}$$ (2)

where **c**_*t*_ represents the centre of the structure at time *t*, **r**_*t*_ represents the elements of a reference frame **R**_*t*_ given by:

$$\begin{array}{c} R_{t}=[\begin{array}{ccc} r_{11_{t}} & r_{12_{t}} & r_{13_{t}}\\ r_{21_{t}} & r_{22_{t}} & r_{23_{t}}\\ r_{31_{t}} & r_{32_{t}} & r_{33_{t}} \end{array}], \end{array}$$ (3)

giving a global orientation, and **e**_*t*_ represents the ellipse parameters comprising the length of the ellipse major *a*_*t*_ and minor *b*_*t*_ axes, as well as the orientation *ϕ*_*t*_ relative to reference frame **R**.

### 2.4 Prediction Model

The state prediction is modelled as an addition of the normal vector to the position vector with the addition of a zero mean Gaussian noise vector **ϵ**_*c*_:

$$\begin{array}{c} c_{t}=c_{t-1}+{\left[r_{13_{t}}\;r_{23_{t}}\;r_{33_{t}}\right]}^{T}+\epsilon_{c}. \end{array}$$ (4)

Similarly, the ellipse parameters are corrupted by Gaussian noise vector **ϵ**_*e*_:

$$\begin{array}{c} e_{t}=e_{t-1}+\epsilon_{e}. \end{array}$$ (5)

The reference frame is however perturbed by a function whose behaviour is defined in the following section:

$$\begin{array}{c} R_{t}=f_{n}\left(R_{t-1},\epsilon_{r}\right) \end{array}$$ (6)

#### 2.4.1 Normal Vector Perturbation

Without lack of generality, the mean orientation of the normal noise vector can be considered to be in the direction (0, 0, 1). This can be represented in terms of spherical coordinates, with a polar angle *ϵ*_*θ*_ and azimuth *ϵ*_*ψ*_ as:

$$\begin{array}{c} \epsilon_{r}={\left[\text{cos}\epsilon_{\theta }\text{sin}\epsilon_{\psi }\;\;\text{sin}\epsilon_{\theta }\text{sin}\epsilon_{\psi }\;\;\text{cos}\epsilon_{\psi }\right]}^{T}. \end{array}$$ (7)

**Fig 2:**
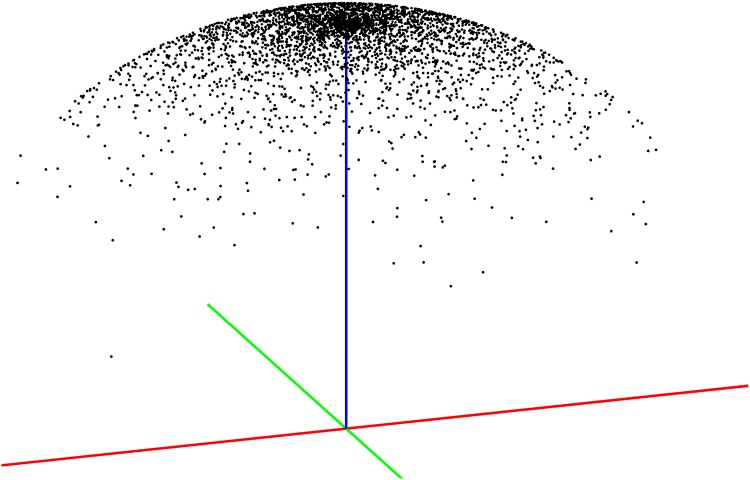
Perturbation of the state normal vector from a uniform azimuth and normally distributed polar angle. Here, a set of example samples are displayed as black markers, about the previous normal vector displayed in blue.

*ϵ*_*θ*_ can be sampled from the uniform distribution (displayed in Fig 2):

$$\begin{array}{c} \epsilon_{\theta }∼U\left(0,2\pi \right), \end{array}$$ (8)

and *ϵ*_*ψ*_ is normally distributed around 0, such that the samples are more sparse the further the angle from the pole:

$$\begin{array}{c} \epsilon_{\psi }∼N\left(0,\sigma_{\psi }^{2}\right). \end{array}$$ (9)

With the assumption that the particle position **c**^**′**^_*t*−1_ = [0 0 1]^*T*^ and **c**^**′**^_*t*_ = [*cosϵ*_*θ*_*sinϵ*_*ψ*_ *sinϵ*_*θ*_*sinϵ*_*ψ*_ *cosϵ*_*ψ*_]^*T*^ and rotation frames:

$$\begin{array}{c} R_{t-1}^{\prime }=[\begin{array}{ccc} 1 & 0 & 0\\ 0 & 1 & 0\\ 0 & 0 & 1 \end{array}],\;R_{t}^{\prime }=[\begin{array}{ccc} \phi_{a} & \phi_{b} & \text{cos}\epsilon_{\theta }\;\text{sin}\epsilon_{\psi }\\ \phi_{c} & \phi_{d} & \text{sin}\epsilon_{\theta }\;\text{sin}\epsilon_{\psi }\\ \phi_{e} & \phi_{f} & \text{cos}\epsilon_{\psi } \end{array}], \end{array}$$ (10)

the variables *ϕ*_*a*_, …, *ϕ*_*f*_ of reference frame **R**^**′**^_*t*_ that minimise the twist between frame **R**^**′**^_*t*−1_ and **R**^**′**^_*t*_ can be found by sweeping a Rotation Minimizing Frame (RMF) through points **c**^**′**^_*t*−1_ and **c**^**′**^_*t*_ using the double reflection method in [24]. This ‘local’ reference frame **R**^**′**^_*t*_ can be used to update the global reference frame **R**_*t*_ in the state vector using:

$$\begin{array}{c} R_{t}=R_{t-1}R_{t}^{\prime }. \end{array}$$ (11)

### 2.5 Measurement Model

**Fig 3:**
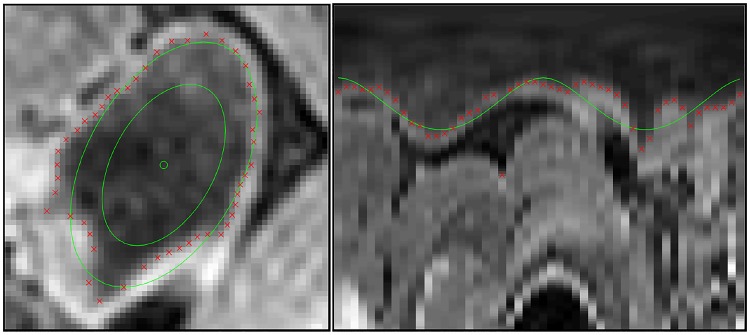
Measurement of a single state visualised in both crosssection view (left) and radial coordinates (right). Ellipse parameters are visualised in green, and detected colon wall points are marked in red.

The proposed model uses cross-sectional radial intensity profiles to assign a probability of the observed data **z**_*t*_ given a state **x**_*t*_. We use the approximation that the colon is topologically cylindrical, with an elliptical cross-section of varying parameters (see Fig 3). The assumption is made that the intra-luminal space has a low local intensity, relative to the bowel wall, due to the ingested contrast agent. At the position of the colon wall, a rapid increase in the image intensity is observed. It is this increase in image intensity that is detected across the radial intensity profile by defining an intensity threshold. The likelihood function *p*(**z**_*t*_|**x**_*t*_) is then defined using the proximity of the detected boundary points to the predicted ellipse. The ellipse boundary positions **u** are defined as:

$$\begin{array}{c} u_{t}^{h}=R_{t}[\begin{array}{c} a_{t}\text{cos}\left(h\right)\text{cos}\left(\phi_{t}\right)-b_{t}\text{sin}\left(h\right)\text{sin}\left(\phi \right)\\ a_{t}\text{cos}\left(h\right)\text{sin}\left(\phi_{t}\right)+b_{t}\text{sin}\left(h\right)\text{cos}\left(\phi \right)\\ 0 \end{array}]+c_{t}, \end{array}$$ (12)

where *h* ∼ [0, 2*π*] and *H* is the number of sampled positions. Similarly, the unit vector from **c**_*t*_ to each point in **u** is used to interpolate a radial intensity profile and detect the bowel wall given by an intensity value exceeding some threshold *k*. A term is included to penalise states in which bowel wall is detected within an inner ellipse defined by:

$$\begin{array}{c} u_{inner_{t}}^{h}=R_{t}(\eta [\begin{array}{c} a_{t}\text{cos}\left(h\right)\text{cos}\left(\phi_{t}\right)-b_{t}\text{sin}\left(h\right)\text{sin}\left(\phi \right)\\ a_{t}\text{cos}\left(h\right)\text{sin}\left(\phi_{t}\right)+b_{t}\text{sin}\left(h\right)\text{cos}\left(\phi \right)\\ 0 \end{array}])+c_{t}, \end{array}$$ (13)

designed to further discourage crossing of the bowel wall. Lastly, some boundary points may not be detected, and so these are excluded from the measurement model calculation, but incur an additional penalty based on the percentage of missing boundary points. A cost function is then defined as the mean sum of squared distances between the detected boundary points **v** and the ellipse **u**:

$$\begin{array}{c} F_{v,u}\left(v_{t}^{h^{i}},u_{t}^{h^{i}}\right)=\frac{\sum_{h=0}^{2\pi }\left|\right|v_{t}^{h^{i}}-u_{t}^{h^{i}}\left|\right|}{H_{detected}}. \end{array}$$ (14)

where *H*_*detected*_ is the number of detected boundary points. The full measurement model is then:

$$\begin{array}{c} d\left(z_{t}|x_{t}^{i}\right)=\frac{F_{v,u}\left(v_{t}^{h^{i}},u_{t}^{h^{i}}\right)}{(1-ϒ)\Omega }, \end{array}$$ (15)

where *Υ* is the percentage of boundary points detected within inner ellipse $u_{inner_{t}}^{h}$, and Ω is the proportion of detected boundary points. The particle weights can be calculated:

$$\begin{array}{c} q_{k}^{i}\propto q_{k-1}^{i}\frac{\text{exp}(-Wd\left(z_{t}|x_{t}^{i}\right))}{\sum_{m=1}^{N_{s}}\text{exp}(-Wd\left(z_{t}|x_{t}^{m}\right)}, \end{array}$$ (16)

where *W* is a normalising constant and controls the sharpness of the weighting distribution.

The particle filter can be set to run to a maximum number of iterations *T*, or another termination criterion set such that a high rate of particle degeneracy is detected [25]. Here, a small effective sample size $\hat{N_{eff}}$ indicates a high variance in the particle weights **q**^*i*^ and in turn indicates an unlikely state to represent the tracked bowel. This usually occurs due to local colonic collapse, large amounts of high intensity intra-luminal matter, or poor image quality due to image artefact or poor bowel preparation. For this study, termination occurs when $\hat{N_{eff}}$ is less than 1% of the original particle count.

#### 2.5.1 Dynamic bowel wall intensity threshold

For robust tracking of the bowel wall, the wall intensity threshold *k* must be dynamically updated due to the local intensity inhomogeneity caused by the MRI bias field, and also the wall intensity inhomogeneity due to variations in contrast uptake. After each iteration *t* of the particle filter, voxel intensity values are sampled from the position of each detected inner wall position **P**, along the vector which intersects this position and the position of the centreline **C**_**t**_. The voxel intensity values are split into two groups, the wall voxels **V**^**w**^ which start at wall position **P** and follow vector $\vec{C_{t}P}$; and the lumen voxels **V**^**l**^ which follow vector $\vec{PC_{t}}$. The distance sampled along each vector is set at 3*mm* as an approximate bowel wall thickness. Threshold *k* is found by linear search, such that it maximises the separation between these two groups:

$$\begin{array}{c} k=\arg\max_{k}[\tau \sum_{i\in V^{w}}f_{>}\left(V_{i}^{w},k\right)+\left(1-\tau \right)\sum_{i\in V^{l}}f_{<}\left(V_{i}^{l},k\right)], \end{array}$$ (17)

where

$$\begin{array}{c} f_{>}\left(x,k\right)=\{\begin{array}{cc} 1 & \;\text{if}\;x>k\\ 0 & \;\text{otherwise} \end{array}, \end{array}$$ (18)

and

$$\begin{array}{c} f_{<}\left(x,k\right)=\{\begin{array}{cc} 1 & \;\text{if}\;x<k\\ 0 & \;\text{otherwise} \end{array}. \end{array}$$ (19)

#### 2.5.2 Extraction of centreline

**Fig 4:**
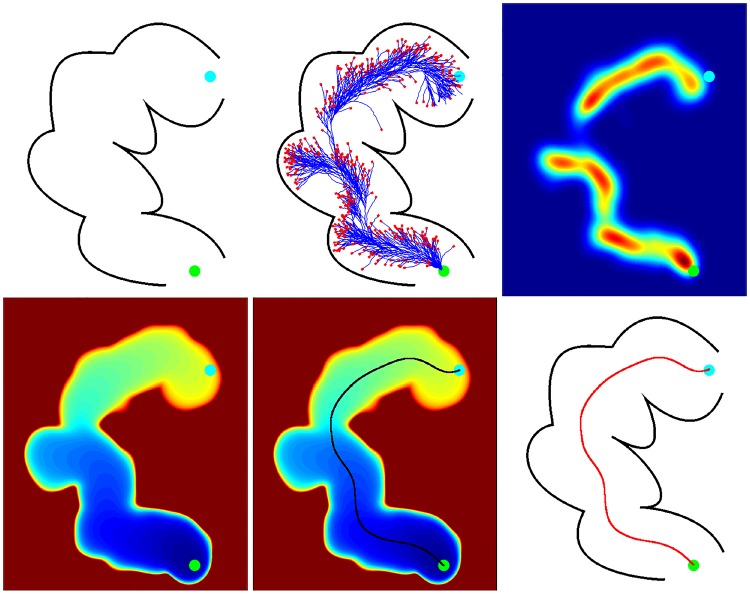
An overview of the particle filtering method. Top row shows: a segment of colon with particle filter start and end points in green and cyan respectively (left); execution of a simplified version of the particle filtering method showing ‘live’ and ‘terminated’ particles in blue and red respectively (middle); the speed function calculated from the weighted sum of Gaussians of particle positions (right). Bottom row shows: the geodesic distances from the start point, calculated by fast marching (left); the centreline path found by computing the shortest geodesic distance from start to end point (middle); the centreline path overlay on the original image (right).

**Fig 5:**
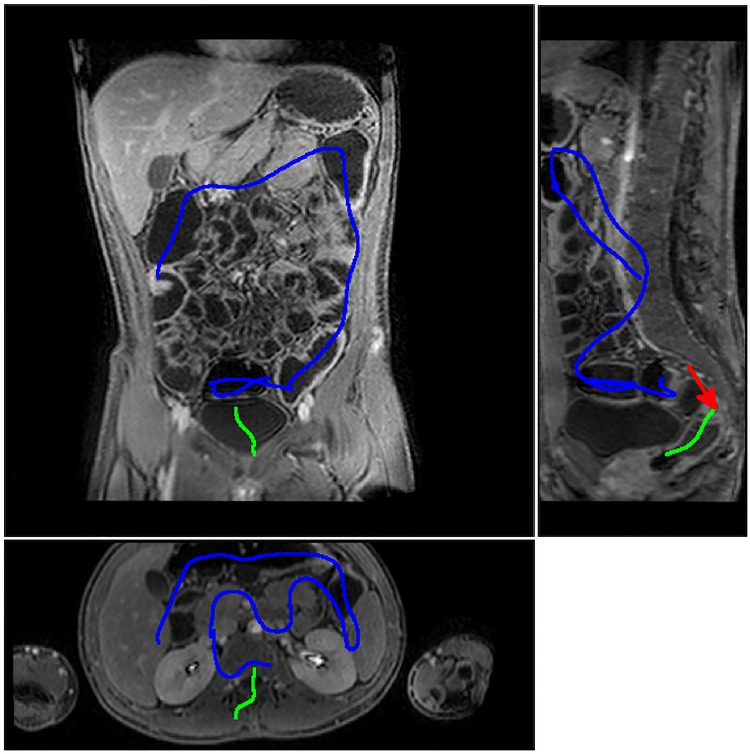
Particle filtering method applied to a patient dataset. The particle filter is first manually initialised in the rectum and tracks the colon to the sigmoid junction (green line) where it is unable to progress due to an area of local colonic collapse (indicated by the red arrow). The algorithm is manually reinitialised at the other side of the collapse and is able to track the colon through to the caecum (blue line). The figure shows both of these three dimensional lines projected onto a single coronal (top left), sagittal (top right) and axial (bottom) plane, and therefore travels in, and out of plane.

After the termination of the particle filter, a centreline is extracted to allow for subsequent measurement of the bowel wall thickness. A simple averaging of the particle centreline position at each iteration **C**_**t**_ gives unsatisfactory results in circumstances where the particle filter explores multiple pathways, as a mean position may not necessarily lie within the colon lumen. Instead, a speed function is derived from the particle positions at every iteration, and fast marching used to find the fastest geodesic path from start to end point. Speed function *G* can be created by computing the weighted sum of Gaussians:

$$\begin{array}{c} G\left(y_{j}\right)=\sum_{i=1}^{N}q_{i}e^{{\left|\right|y_{j}-x_{i}\left|\right|}^{2}/h^{2}} \end{array}$$ (20)

where {*x*_*i*_}_*i*=1,…,*N*_ are the centres of the Gaussians, taken from the centreline positions of each particle state over all iterations; *q*_*i*_ are the weight coefficients taking from the respective particle weights; and *h* is the bandwidth parameter of the Gaussians (set experimentally to 3*mm*). The sum of Gaussians is evaluated at each of the target points {*y*_*j*_}_*j*=1,…,*M*_, in this case the voxel coordinates of the input image domain in $R^{3}$. This can be computed efficiently using a fast Gauss transform function [26]. The centreline can then be extracted by evolving a wave front across the image domain using the fast marching method [27], solving the Eikonal equation:

$$\begin{array}{c} G(x)|\nabla T(x)|=1, \end{array}$$ (21)

where *T* is time. Centreline path is then found by computing the shortest geodesic distance across the resulting distance map from start to end point. A simplified simulation of this workflow is demonstrated in Fig 4 and an example result displayed in Fig 5.

### 2.6 Wall thickness calculation

This section will describe the algorithm used to calculate the bowel wall thickness given a centreline path. To allow for a reference frame to be constructed at each position along the centreline **C**, a Rotation Minimising Frame (RMF) [28] is employed. The properties of such a set of reference frames are such that the magnitude of angles between the reference vectors of consecutive frames *U*_*i*_ *U*_*i*+1_ are minimised, minimising the total global error:

$$\begin{array}{c} E_{g}=\sum_{i=0}^{n-1}\left|∠\left(U_{i},U_{i+1}\right)\right|. \end{array}$$ (22)

Each frame can be defined by its components:

$$\begin{array}{c} U=[r\;s\;t], \end{array}$$ (23)

where **t** defines the tangent vector, and **r** and **s** span the curve normal plane.

Now for any position along the centreline, it is assumed that there exists a full circumferential loop of bowel wall that lies in the image plane in which **r** and **s** lie. To more easily specify a point in this plane, a radial coordinate system is used, parametrised by *θ*, the angle of rotation from **r**; and *f*, the distance from **C**. As the bowel wall is a continuous structure, for each angle of rotation *θ* there exists a position of the inner bowel wall and so the task can be formulated as a labelling problem.

For every frame *U*_*t*_ there exists a set of sites **S**_*t*_ = {1_*t*_, …, *n*_*t*_} which correspond to the angle of rotation around the frame tangent. A set of random variables **F**_*t*_ = {*F*_1_*t*__, …, *F*_*n*_*t*__} which take a label corresponding to a distance from the frame centre **f**_*t*_ = {*f*_1_*t*__, …, *f*_*n*_*t*__}. A neighbourhood system defines edges and allow the definition of connections between sites **N**_*t*_ = {*N*_*i*_*t*__|∀*i*_*t*_ ∈ **S**_*t*_}. Lastly a pairwise clique allows the modelling of the geometric and appearance based dependences between label configurations $C_{2}=\left\{\left\{i_{t},i_{t}^{\prime }\right\}|i_{t}\in S_{t},i_{t}^{\prime }\in N_{i_{t}}\right\}$.

**Fig 6:**
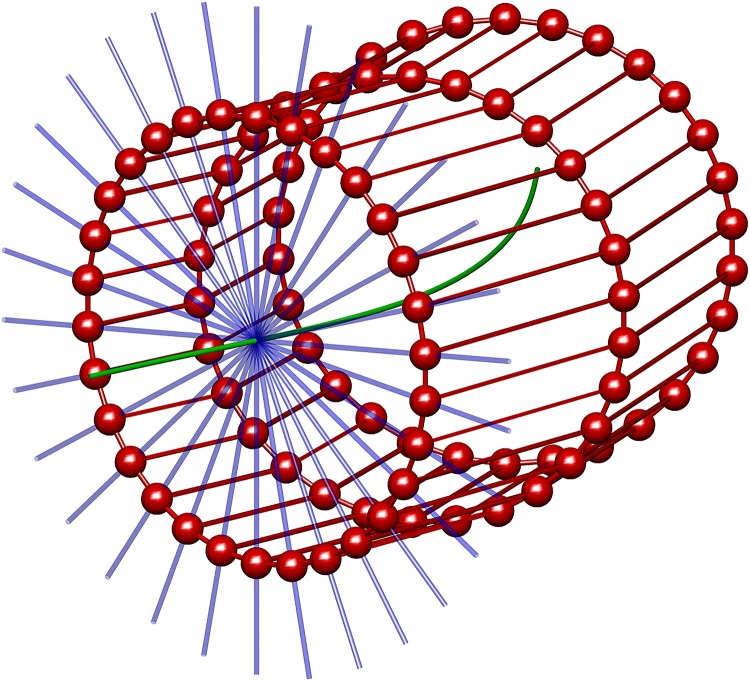
A simplified example of the graph structure described in section 2.6. At regular intervals along the centreline (green), a reference frame is extracted. In the image plane defined by this reference frame, a set of possible site locations are calculated by a radial coordinate system (blue). The set of sites corresponding to each reference frame may lie at any point along this ray. Here, for simplicity, the set of possible site locations for single a frame are shown, although the algorithm optimises over multiple frames. The sites are displayed as red spheres, connected by edges which describe their neighbourhood system. Neighbouring sets of rings correspond to neighbouring reference frames.

Two types of edges are included within the model. The first allows inclusion of dependencies between neighbouring sites within the same frame and also between neighbouring frames *N*_*i*_*t*__ = {(*i* − 1)_*t*_, (*i* + 1)_*t*_, *i*_*t*−1_, *i*_*t*+1_} (see Fig 6 for details).

#### 2.6.1 Node Potentials - Inner Bowel Wall

**Fig 7:**
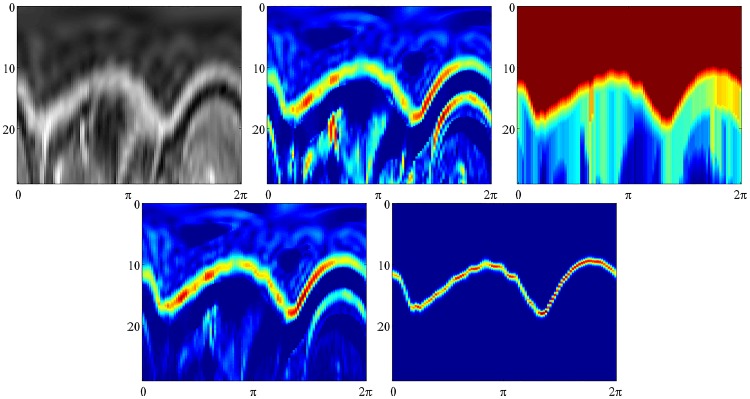
Figures show the construction of node potentials for the detection of the inner bowel wall. All figures are displayed in radial coordinates, with the distance from origin shown in the vertical axis, and the angle of rotation in the horizontal axis. The original image is shown top left. Top middle shows the gradient node potentials $H_{i}^{1}$. Top right shows the cumulative node potentials $H_{i}^{2}$. Bottom left shows the combined node potentials *V*_*i*_. Bottom right shows the belief vectors *b*_*j*_. All measures are scale invariant.

The node potentials (see Fig 7) give a probability of assigning a label *f*_*i*_ to a site *i*. At the position of the inner bowel wall *p*_*i*_ at site *i*, a rapid change of the intensity of the image is expected, and the direction of this gradient should coincide with the unit vector $\hat{Cp_{i}}=\vec{Cp_{i}}/\left|Cp_{i}\right|$ from the frame centre to the site position. The following potentials are defined:

$$\begin{array}{c} H_{i}^{1}\left(i\right)=\{\begin{array}{cc} \left|\nabla I(p_{i})\right| & \;\text{if}\;\hat{\nabla I(p_{i})}\cdot \hat{Cp_{i}}>0\\ |\nabla I\left(p_{i}\right)|{\left(\hat{\nabla I(p_{i})}\cdot \hat{Cp_{i}}+1\right)}^{2} & \;\text{otherwise} \end{array} \end{array}$$ (24)

This function penalises against selecting inner wall positions which have a gradient direction greater than ±*π*/4 radians away from $\hat{Cp_{i}}$. Simply using the image gradient may produce some undesirable results when high contrast artefacts lie outside of the bowel wall, such as blood vessels or when two bowel walls are closely applied to each other. To prevent this from occurring, the result is scaled by the cumulative sum of voxel values from image *I* which lie above threshold *k* (the threshold which maximises the separation between the wall, and lumen voxels—see section 2.5.1), originating from the frame centre **C** along vector $\hat{Cp_{i}}$:

$$\begin{array}{c} H_{i}^{2}\left(i\right)=\frac{\beta }{\sum_{n=0}^{f_{i}}f_{>}\left(I\left(C+n\;\hat{Cp_{i}}\right),k\right)}, \end{array}$$ (25)

where

$$\begin{array}{c} f_{>}\left(x,k\right)=\{\begin{array}{cc} 1 & \;\text{if}\;x>k\\ 0 & \;\text{otherwise} \end{array}. \end{array}$$ (26)

and so the full site potential equation is:

$$\begin{array}{c} V_{i}\left(i\right)=H_{i}^{1}\left(i\right)H_{i}^{2}\left(i\right). \end{array}$$ (27)

#### 2.6.2 Edge potentials - Inner Bowel Wall

The solution is constrained such that neighbouring sites must be both a similar distance to the centre of the frame, and have a similar voxel intensity value. This gives a result that is both continuous in terms of the bowel wall position, and the intensity of the signal at those positions. To model this, the following edge potentials are used:

$$\begin{array}{c} V_{i,j}\left(i,j\right)=e^{-\gamma ||f_{i}-f_{j}\left|\right|}{\left(1-\frac{\min(I\left(p_{i}\right),I\left(p_{j}\right))}{\max(I\left(p_{i}\right),I\left(p_{j}\right))}\right)}^{2}, \end{array}$$ (28)

where *f*_*i*_ is the distance of site *i* from the frame centre.

#### 2.6.3 Belief Propagation Inference

We wish to find the most probable solution of label assignments.

$$\begin{array}{c} Pr\left(f\right)=\frac{1}{Z}\prod_{i\in S}V_{i}\left(f_{i}\right)\prod_{i\in S}\prod_{j\in N_{i}}V_{ij}\left(f_{i},f_{j}\right), \end{array}$$ (29)

Z is intractable, but we can use the Max-product Belief Propagation (BP) algorithm to find the optimal global solution [29]:

$$\begin{array}{c} f^{\left(MAP\right)}=\arg\max_{f}[\prod_{i\in S}V_{i}\left(f_{i}\right)\prod_{i\in S}\prod_{j\in N_{i}}V_{ij}\left(f_{i},f_{j}\right)], \end{array}$$ (30)

It is known that BP is exact on acyclic tree-like graphical models, but has been shown to give a good MAP estimate in graphs with loops. The BP algorithm works by passing messages between nodes of a graph defined by the set of sites **θ**, with edges defined by the site neighbourhoods **N**. Each message *M* is an *i* dimensional vector, with *i* equal to the number of possible labels. At each iteration at time *t*, every node sends messages to each of its neighbours in parallel, whilst also receiving messages itself. Let $m_{p\to q}^{t}$ be the message that node *p* passes to node *q* at iteration *t*. All entries in $m_{p}^{0}\to q$ are initialised to zero. At each iteration, new messages are computed as follows:

$$\begin{array}{c} m_{i\to j}^{t}\left(f_{j}\right)=\max_{f_{i}}(V_{i}\left(f_{i}\right)V_{ij}\left(f_{i},f_{j}\right)\prod_{s\in N_{i}\backslash j}m_{s\to i}^{t-1}\left(f_{i}\right)), \end{array}$$ (31)

where *N*_*i*_∖*j* denotes all neighbours of *i* other than *j*. After *T* iterations, the belief vector for each node may be computed:

$$\begin{array}{c} b_{j}\left(f_{j}\right)=[V_{j}\left(f_{j}\right)\prod_{s\in N_{j}}m_{s\to j}^{T}\left(f_{j}\right)]. \end{array}$$ (32)

The belief vector *b*_*j*_(*f*_*j*_) expresses the relative probability of assigning each label *f*_*j*_ to site *j*. Once the algorithm has terminated, each node is assigned the label having the maximum belief:

$$\begin{array}{c} f_{q}^{*}=\arg\max_{f_{q}\in f}b_{q}\left(f_{q}\right). \end{array}$$ (33)

### 2.7 Calculation of the outer wall position

Following the completion of the BP algorithm, the MAP solution for the set of sites can be used to create a contour marking the position of the inner bowel wall. It can be assumed that there are a set of positions that lie along the normal vectors of this contour, that will also form a closed contour that coincides with the position of the outer bowel wall. Therefore, a similar protocol as described in the previous section can be followed, with the primary change being that the outer bowel wall positions are to be located relative to the inner bowel wall positions. However there are also changes made to the cost functions used.

#### 2.7.1 Node potentials - Outer Bowel Wall

**Fig 8:**
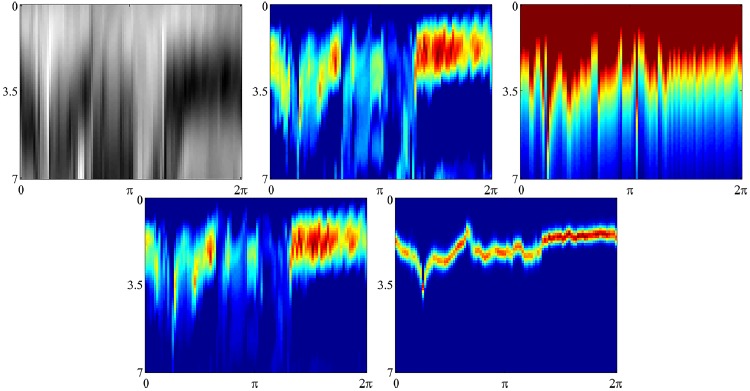
Figures show the construction of node potentials for the detection of the outer bowel wall. All figures are displayed in radial coordinates, with the distance from origin shown in the vertical axis, and the angle of rotation in the horizontal axis. The original image is shown top left. Top middle shows the gradient node potentials $H_{i}^{1}$. Top right shows the cumulative node potentials $H_{i}^{2}$. Bottom left shows the combined node potentials *V*_*i*_. Bottom right shows the belief vectors *b*_*j*_. All measures are scale invariant.

Here, a solution with a rapid decrease in gradient in the direction of the normal of the inner bowel wall contour is desired (see Fig 8). A similar strategy as in Eq (24) is followed. This node potential differs only by the sign in the parentheses in comparison to the inner bowel wall node potential, which takes into account the direction of the gradient:

$$\begin{array}{c} H_{i}^{1}\left(i\right)=\{\begin{array}{cc} \left|\nabla I(p_{i})\right| & \;\text{if}\;\hat{\nabla I(p_{i})}\cdot \hat{Cp_{i}}<0\\ |\nabla I\left(p_{i}\right)|{\left(\hat{\nabla I(p_{i})}\cdot \hat{Cp_{i}}-1\right)}^{2} & \;\text{otherwise} \end{array}. \end{array}$$ (34)

Similarly to the node potentials for the inner bowel wall, we wish to prevent a high potential value being assigned to high contrast artefacts which lie outside the bowel wall. For each site *i*, the position *g*_*i*_ of the first local minima in image gradient can be found along the vector from the frame centre to site $\hat{C_{p_{i}}}$.

$$\begin{array}{c} H_{i}^{2}\left(i\right)=\frac{\kappa }{F(p_{i})}, \end{array}$$ (35)

where

$$\begin{array}{c} F\left(p_{i}\right)=\{\begin{array}{cc} 1 & \;\text{if}\;p_{i}=g_{i}\\ F(p_{i-1})+1 & \;\text{if}\;F(p_{i-1})>0\\ 0 & \;\text{otherwise} \end{array} \end{array}$$ (36)

#### 2.7.2 Edge potentials - Outer Bowel Wall

**Fig 9:**
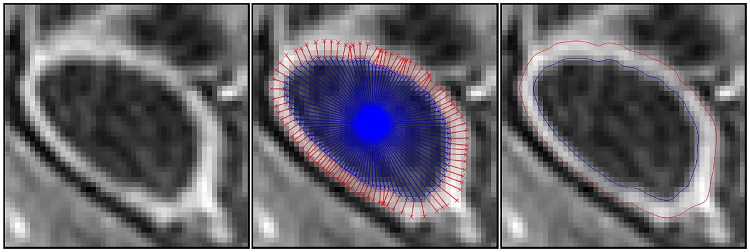
Overview of the wall thickness measurement. A cross-section of the bowel is taken (left), the inner- and outer-walls are detected (centre) and the final contour for both is generated (right).

Similar pair-wise constraints are applied to the outer as to the inner bowel wall for neighbouring sites. The solution is constrained so that the sites are a similar distance from the contour that defines the inner bowel wall, and the intensity of the signal at those positions must be similar. The same potentials as in Eq (28) are used. The label assignments are found using belief propagation. An example result for the wall thickness measurement is shown in Fig 9.

### 2.8 Algorithm Initialisation

**Fig 10:**
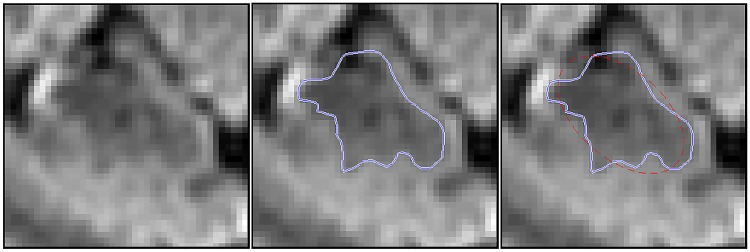
Using the GUI to specify the ellipse parameters for the initial particle filter state. The centre image shows the freehand drawn inner bowel wall contour (solid blue line), and the right image shows the least-square fit ellipse (red dashed line).

The particle filter is initialised by setting all particles with the same parameters, therefore an initial state must be defined (see Eq (2)). The centre point parameters [*c*_*x*_1__ *c*_*y*_1__ *c*_*z*_1__]^*T*^ are selected with the use of a DICOM viewer to give a position in Euclidean space. The reference frame **R**_**1**_ is set such that the tangent [*r*_13_1__ *r*_23_1__ *r*_33_1__]^*T*^ is oriented along the colon lumen, and the other two frame vectors are set at arbitrary, orthogonal angles in the normal plane. The ellipse parameters [*a*_1_ *b*_1_ *ϕ*_1_]^*T*^ are set by a semiautomatic method. First the volume is sampled along the intersecting plane defined by the normal vectors of *R*_1_, to create the resliced image *I*_*r*_ spanning the normal plane. A graphical user interface (GUI) (see Fig 10) allows selection of points *z* = [*x* *y*] in $R^{2}$ defined by **R**_**1**_. An ellipse may be fit by least-squares minimisation [30].

### 2.9 Imaging Protocol

Three hours prior to the scan, participants were requested to drink 3L of 2% mannitol (Baxter, UK) solution to distend the bowel and add contrast. Each patient was cannulated (22G, Introcan Safety, Braun) into the antecubital vein prior to lying in the scanner (3T Philips Achieva, Philips Healthcare, Best, The Netherlands). Each subject was scanned in the prone position with routine anatomical MRI scans acquired following spasmolysis with butyscopolamine (20mg Buscopan, Boehringer Ingelheim).

For this study a 15s breath-hold 3D post-gadolinium contrast (0.1 ml/kg Gadovist 1.0mmol/ml, Bayer Schering Pharma, Berlin, Germany)—T1 high resolution isotropic volume excitation (THRIVE) sequence was used. Each participant had 90 coronal slices with the following parameters: spatial resolution = 2 × 2 × 2*mm*, TR = 2.18*ms*, TE = 1.02*ms*, Averages = 1, acquisition matrix = 228 × 223, flip angle = 10 degrees using the manufacturer’s torso coil.

### 2.10 Ethics Statement

This research was conducted following approval from the North Hampstead Research Ethics Committee (10/H0720/91, 22/12/2010) and sponsored by University College London Hospitals. Approval, on behalf of the participants, was granted by the Research Ethics Committee to use a limited amount of their anonymised data within UCL Centre for Medical Imaging and selected partner organisation groups for research purposes only.

### 2.11 Patient Demographics

24 participants with an existing diagnosis of Crohn’s disease based on a combination of clinical, biochemical, endoscopic, imaging and histopathological findings were identified (8 male) with the mean age 31.3 years (range 19 to 64).

### 2.12 Experimental Design

**Fig 11:**
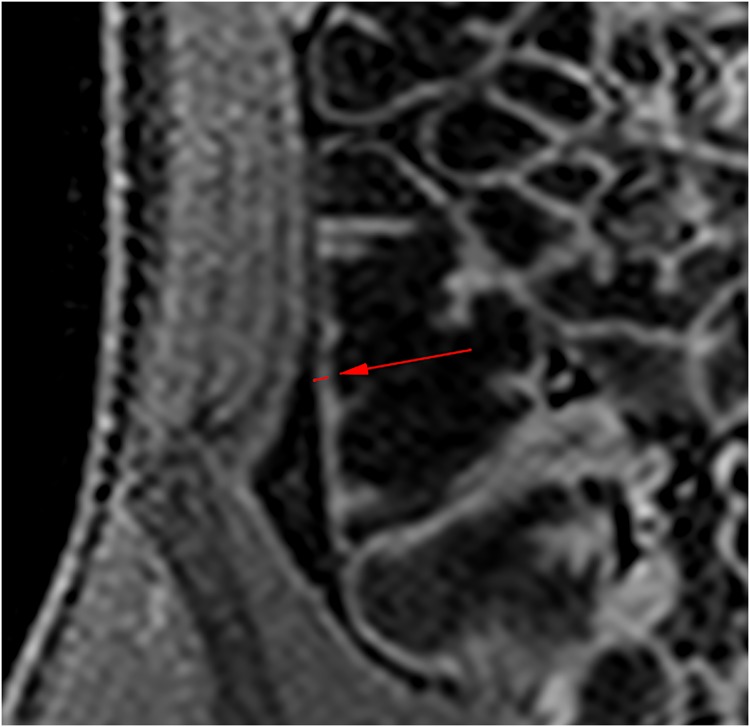
Placements of the ROIs were first selected by the study coordinator, and the approximate position marked with an arrow, pointing towards the bowel wall. The observers independently created a measurement of the bowel wall thickness from an inner bowel wall position that they thought best corresponded with the arrow. This was to minimise the bias on wall thickness measurement induced by the arrow marker.

For the purpose of establishing the accuracy of the bowel wall thickness measurement algorithm, 24 patient cases were chosen at random and without exclusion. In each case, between 6 and 8 locations were selected, evenly distributed along the large bowel from rectum to caecum, by arrows pointing in the direction of the inner bowel wall. Two radiologists (AA, GB) independently created a second set of ROIs by measuring the thickness of the bowel wall from a point on the inner bowel wall which has been identified by the study coordinator (AM), such that it is closest to the preselected location (see Fig 11). 4 patient cases were duplicated to allow an estimation of intra-observer error and kept blind to the radiologists. The data was assessed non-consecutively and randomised by the study coordinator, using a pseudorandom number generator. The algorithm described above was executed on each of the cases by selecting a start and end point in the centre of the bowel lumen at ≈10*cm* distance along the length of the bowel. To allow for a comparison against the observers’ measurements, for each ROI, the thickness measurement where the inner wall position has the smallest Euclidean distance to the observer measurement is selected. If there was no measurement, or the closest measured point was >5*mm* to the observer ROI, the algorithm is said to have failed to make that measurement.

## 3 Results

### 3.1 Overview of results

**Fig 12:**
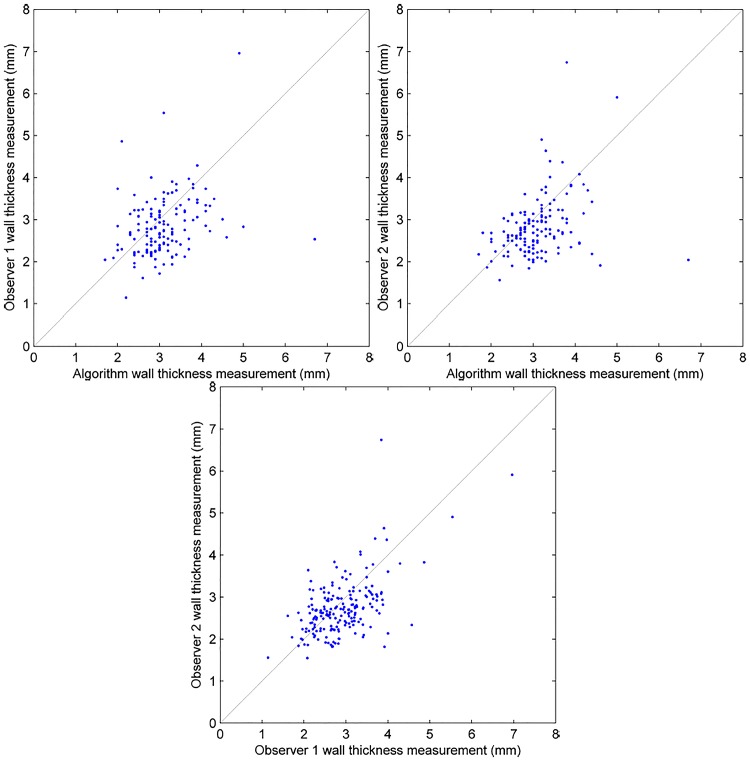
Agreement between the algorithm wall thickness measurements and observer 1 (top left), observer 2 (top right) and a comparison of the individual observer measurements (bottom).

**Fig 13:**
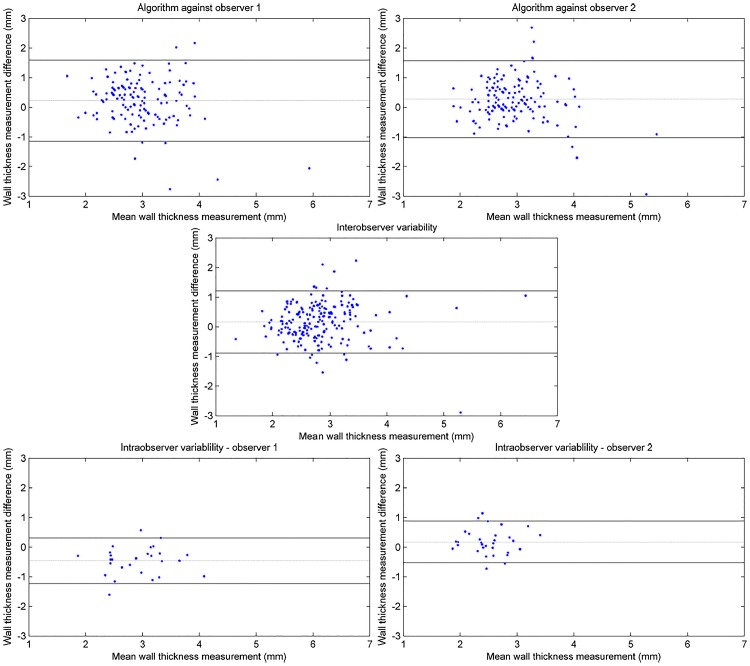
Bland Altman figures showing the variability of wall thickness measurement between: the algorithm and the individual observers (top), the two observers (middle), the observers and their repeat measurements (bottom). The broken line indicates the mean measurement difference. Solid lines indicate the 95% limits of agreement.

**Fig 14:**
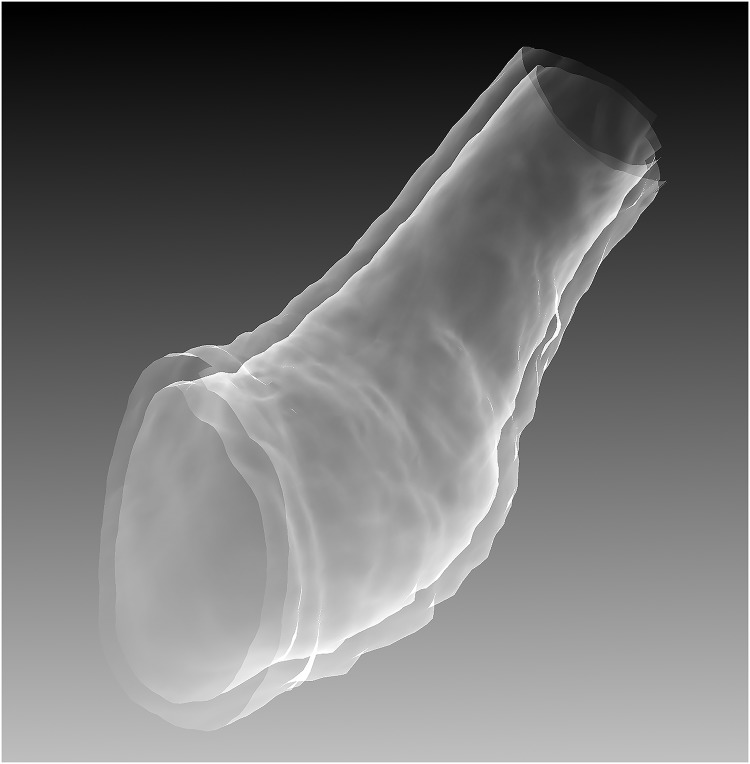
A short (80mm) section of stricture in the descending colon of case 22. The inner and outer bowel walls are reconstructed from the points detected by the algorithm.

The results showing the comparison of wall thickness measurements between the algorithm and the individual observer (Fig 12), as well as the level of inter- and intra-observer variability are show in Table 1, and also in the Bland-Altman plots [31] (Fig 13). It can be seen that, in the ROIs which were successfully measured by the algorithm, the mean difference in wall thickness measurement, and the standard deviation of those differences, between the algorithm and the individual observer (mean difference 0.23*mm* − 0.27*mm*, standard deviation ±0.83*mm* − ±0.79*mm*) were comparable to that of the intra-observer variability (mean difference 0.16*mm*, standard deviation ±0.64*mm*). It can also be seen that the distribution of differences of wall thickness measurement is similar, shown in the Bland-Altman plots in Fig 13. However, in around 15% of the ROIs, the algorithm failed to make a measurement within 5*mm* of the same position as the observer. A section of bowel has been reconstructed from the algorithm measurements and shown in Fig 14.

**Table 1:** Variability and mean difference of wall thickness measurement between the algorithm and the individual observers, the two observers and the observers and their repeat measurements.

	Total ROIs	ROIs included	Mean diff. (mm)	St.D (mm)	95% limits of agreement
Algorithm and observer 1	167	141 (84.4%)	0.23mm	± 0.83mm	± 1.37mm
Algorithm and observer 2	167	143 (85.6%)	0.27mm	± 0.79mm	± 1.30mm
Observer 1 and observer 2	167	167 (100.0%)	0.16mm	± 0.64mm	± 1.05mm
Observer 1 repeat measurements	31	31 (100%)	-0.46mm	± 0.47mm	± 0.77mm
Observer 2 repeat measurements	31	31 (100%)	0.18mm	± 0.43mm	± 0.70mm

### 3.2 Reasons for algorithm failure

**Fig 15:**
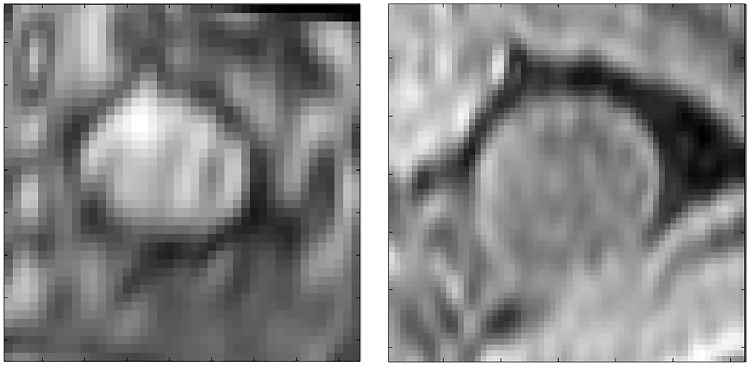
Examples of poor contrast between the lumen and the bowel wall. Each axis ‘tick’ is 1mm.

**Fig 16:**
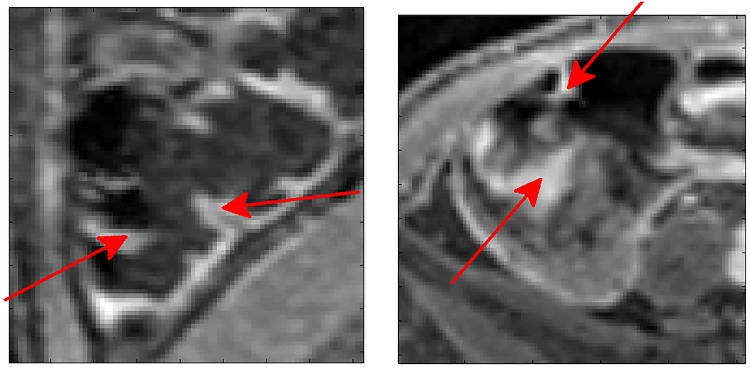
Examples of poorly prepared cases. In both images there is remaining faecal matter which appears as a non homogeneous, high intensity pattern. The image on the left shows an even distribution over the cross section, which makes detecting the bowel wall based on intensity value difficult. The image on the right shows matter (red arrow) floating on the surface of the mannitol solution (image aligned with gravity acting downwards), which may be detected as the location of the bowel wall. Each axis ‘tick’ is 1mm.

**Fig 17:**
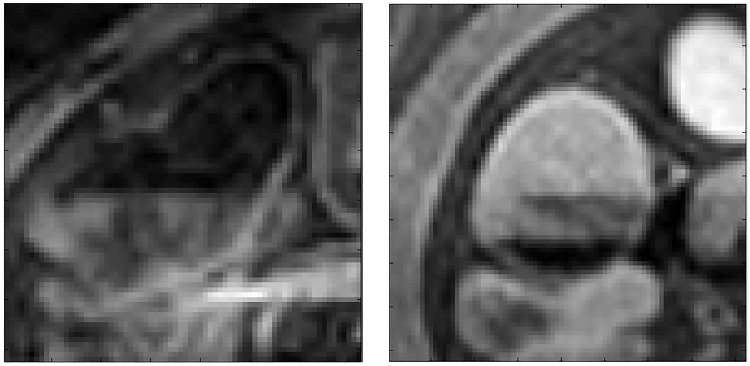
Examples where a strong bias field (left) or reconstruction artefact (right) occurs within a loop of the bowel. As a single intensity value is used for each loop of the bowel wall during the tracking, this strong difference in intensity may result in the incorrect location being detected. Each axis ‘tick’ is 1mm.

**Fig 18:**
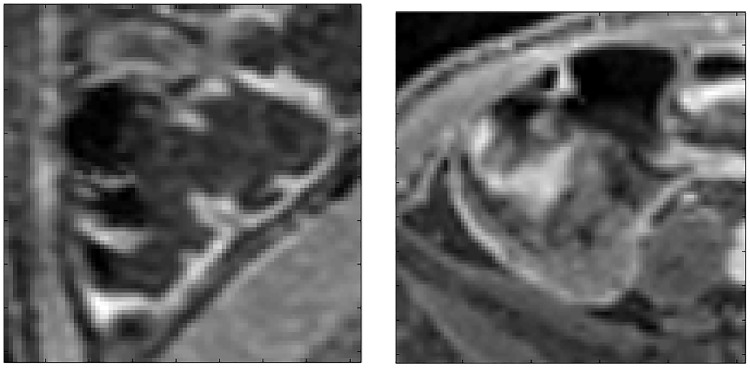
Examples of heavily haustrated regions, indicated by the red arrows, in the descending colon (left) and caecum (right). In these cases more than a single loop of bowel appears in a single cross section, and therefore the incorrect location of the inner bowel wall may be detected. Each axis ‘tick’ is 1mm.

When taking the comparison of the magnitude of wall thickness measurements made by the algorithm, and the measurements made by observers 1 and 2, 26 (15.6%) and 24 (14.4%) out of 167 ROIs respectively were not within 5*mm* of each other. In these cases, the primary reasons and the frequency of this occurrence along with examples of regions used in the experiment are listed below:

- Poor wall contrast (4.8%): These areas showed very poor contrast between the lumen and the bowel wall, resulting in the inner and/or outer wall locations being detected incorrectly. This can also make the particle filter tracking fail (see Fig 15).
- Poor preparation (4.2%): Residual faecal matter in the bowel results in areas of high signal which are indistinguishable in terms of intensity value from the bowel wall. Due to the wall detection being made on image gradient values, this residual matter can result in the incorrect detection of the inner bowel wall (see Fig 16).
- Bias field/reconstruction artefact (2.4%): The algorithm is dynamic, updating any threshold values for bowel wall detection as iterations progress along the centreline; however the assumption is made that for each circumferential bowel wall loop, the wall intensity values are relatively constant. In the majority of cases, any inhomogeneities due to the bias field do not hinder the performance of the algorithm, but in a small number of patient cases a steep gradient is observed (see Fig 17).
- Heavily haustrated (2.4%): Problems due to large haustral folds mainly occurred in the caecum. If the detected centreline did not lie exactly central, a given cross section may be intersected by a large haustral fold. As the algorithm searches for the first bowel wall location from the centreline, the haustral fold may be incorrectly identified (see Fig 18).
- Motion artefact (1.2%): In a small number of cases motion artefacts resulted in a blurred image of the bowel wall and therefore the algorithm failed in detecting it correctly.

## 4 Discussion

The workflow is automated, given a seed point and initial ellipse configuration. Results are promising, showing a similar level of variability between the algorithm and the observer in comparison to the variability between the individual observers; however the algorithm has a slightly lower agreement to the observers. As the method is automated given a manual seed placement, it has the advantage over a human reader that a very large number of wall thickness measurements can be made rather than the relatively small number of manually placed measurements an observer might make. Such information could provide a more accurate mean measurement or allow the reader to compare the variation and distribution of wall thickness. This method also has the potential to make more consistent measurements, something that has been shown to be an issue with the human reader.

However, an obvious disadvantage is the failure of the algorithm in around 15% of cases. This is primarily due to the algorithm’s expectation to detect a full loop of bowel in each cross sectional image. Due to the reasons listed above (poor wall contrast, poor preparation, bias field/reconstruction artefact, heavily haustruated sections, motion artefact), this is not possible using the assumptions made in the methods section of this paper, and therefore further domain specific information would have to be incorporated into the algorithm in order to cope with these use cases. Furthermore, the algorithm does not currently give any indication of the accuracy of a given measurement. On many of the cross sectional images it is difficult to give an accurate measurement, even for the human observer and so quantification of measurement accuracy is important to draw any meaningful clinic value from the results. This would be especially important if one wanted to create a mean thickness measurement along a section of bowel, which could be useful in cases of severe inflammation or stricture. To overcome inaccuracies due to poor preparation which occurs frequently in clinical practice, it may be possible to incorporate textural information to better identify lumen and bowel wall. This approach has been used in automatically detecting areas of Crohn’s disease in MR volumes [32] by machine learning techniques and could be better used to classify voxel type.

In conclusion, we have presented a method for automated bowel wall thickness measurement which has shown similar levels of variability to two observers in 142 ROIs over 24 patient cases. This is an extremely challenging task as the range of locations picked to assess bowel wall thickness were not representative of clinical cases where optimal regions of good distension are expected. Results using clinically representative data may be better. Further work is needed before clinical use. This should address the reasons for algorithm failure which have been identified to be cases with poor wall contrast, poor preparation, bias field/reconstruction artefact, heavily haustruated sections and motion artefact.
